# Supplementary material for: Inhibition of AXL receptor tyrosine kinase enhances brown adipose tissue functionality in mice
Source: Nat Commun. 2023 Jul 13;14:4162. doi: 10.1038/s41467-023-39715-8 (PMC10344962; doi:10.1038/s41467-023-39715-8)
Supplement: Supplementary file 1 — Supplementary Information [file 41467_2023_39715_MOESM1_ESM.pdf]

Inhibition of AXL Receptor Tyrosine Kinase Enhances Brown Adipose Tissue Functionality in mice

Vissarion Efthymiou[1,2#], Lianggong Ding[1#], Miroslav Balaz[1,3], Wenfei Sun[1,4], Lucia Balazova[1,3], Leon G Straub[1,5], Hua Dong[1,6], Eric Simon[7], Adhideb Ghosh[1], Aliki Perdikari[1], Svenja Keller[1,8], Umesh Ghoshdastider[1], Carla Horvath[1], Caroline Moser[1], Bradford Hamilton[9], Heike Neubauer[9], Christian Wolfrum[1*]

*Corresponding Author

Corresponding Author e-mail address: christian-wolfrum@ethz.ch

[#]These two authors share equal contribution

[1]ETH Zürich – Swiss Federal Institute of Technology, Department of Health Sciences and Technology, Laboratory of Translational Nutrition Biology, Institute of Food, Nutrition and Health, Schwerzenbach, Switzerland

[2]Joslin Diabetes Center, Section of Integrative Physiology and Metabolism, Research Division, Harvard Medical School, Boston MA, USA

[3]Laboratory of Cellular and Molecular Metabolism, Biomedical Research Center, Slovak Academy of Sciences, Bratislava, Slovakia

[4]Department of Bioengineering, Stanford University, Stanford, CA, USA; Department of Molecular and Cellular Physiology, Stanford University School of Medicine, Stanford, CA, USA

[5]Institute of Child Nutrition, Max Rubner-Institut, Federal Research Institute of Nutrition and Food, Karlsruhe, Germany

[6]Institute for Stem Cell Biology and Regenerative Medicine, Stanford University School of Medicine, Stanford, CA, USA

[7]Department of Global Computational Biology and Digital Sciences, Boehringer Ingelheim Pharma GmbH & Co. KG, Biberach an der Riss, Germany

[8]Mechanisms of Inherited Kidney Diseases Group, Institute of Physiology, University of Zurich, 8057 Zurich, Switzerland

[9]Department of CardioMetabolic Diseases Research, Boehringer Ingelheim Pharma GmbH & Co. KG, Biberach an der Riss, Germany

Supplementary Figures and Tables

Supplementary Figure 1

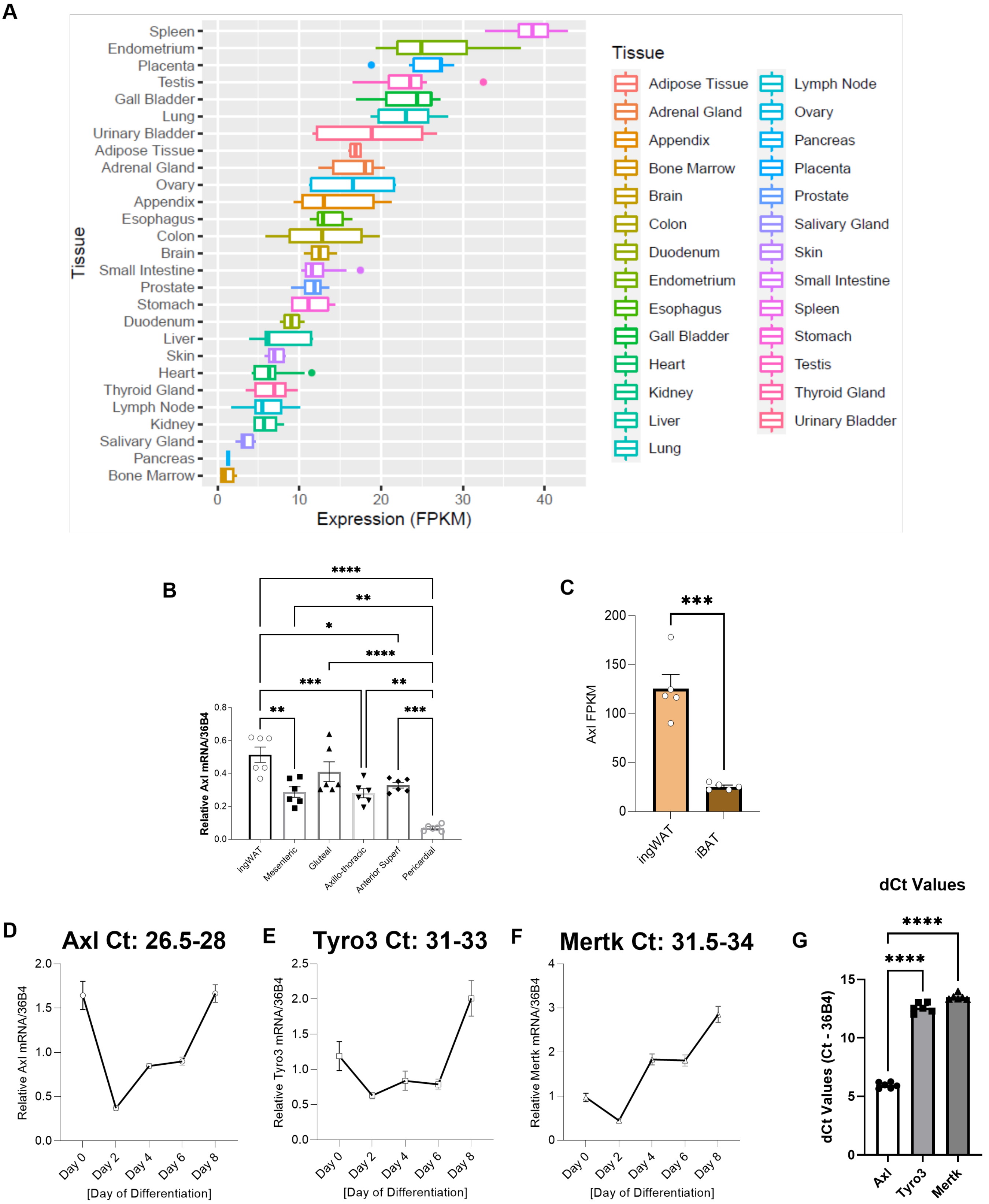

Figure S1: A) RNA sequencing analysis. mRNA expression of AXL Receptor Tyrosine Kinase in tissue panel obtained from human tissues[1]. Box in the plot represents the interquartile range between the 25th and 75th percentiles of the data. Line inside the box represents the median value of the data. The whiskers extending from the box represent the range of the data, excluding any outliers. Outliers are represented as individual points outside the whiskers (Adipose Tissue n=5; Adrenal Gland n=6; Appendix n=6; Bone Marrow n=6; Cerebral Cortex n=3; Colon n=6; Duodenum n=4; Endometrium n=7; Esophagus n=6; Gall Bladder n=7; Heart n=9; Kidney n=4; Liver n=5; Lung n=8; Lymph Node n=13; Ovary n=4; Pancreas n=4; Placenta n=7; Prostate n=7; Salivary Gland n=6; Skin n=6; Small Intestine n=8; Spleen n=5; Stomach n=4; Testis n=8; Thyroid Gland n=9; Urinary Bladder n=6). B) mRNA expression of Axl receptor in panel of WAT depots obtained by ob/ob mice (n=6/group). C) RNA sequencing results (in FPKM) of Axl receptor in ingWAT and iBAT depots obtained from WT C57Bl/6 mice (n=5/group) D-F) mRNA expression of the members of the TAM family (Axl (D), Tyro3 (E), Mertk (F)) in iBAs from day 0 until day 8 of differentiation. Ct values of detection by qPCR are signified above the graphs (n=8/group). G) dCt values for the detection of gene expression for Axl, Tyro3, and Mertk by qPCR (n=6/group). For all graphs, results are presented as average ± SEM ***$p<0.001$, ****$p<0.0001$. For two group comparisons (C) unpaired two-tailed t-test was performed, for three or more group comparisons (B, G) one-way ANOVA was performed (Tukey test was applied to correct for multiple comparisons).

Supplementary Figure 2

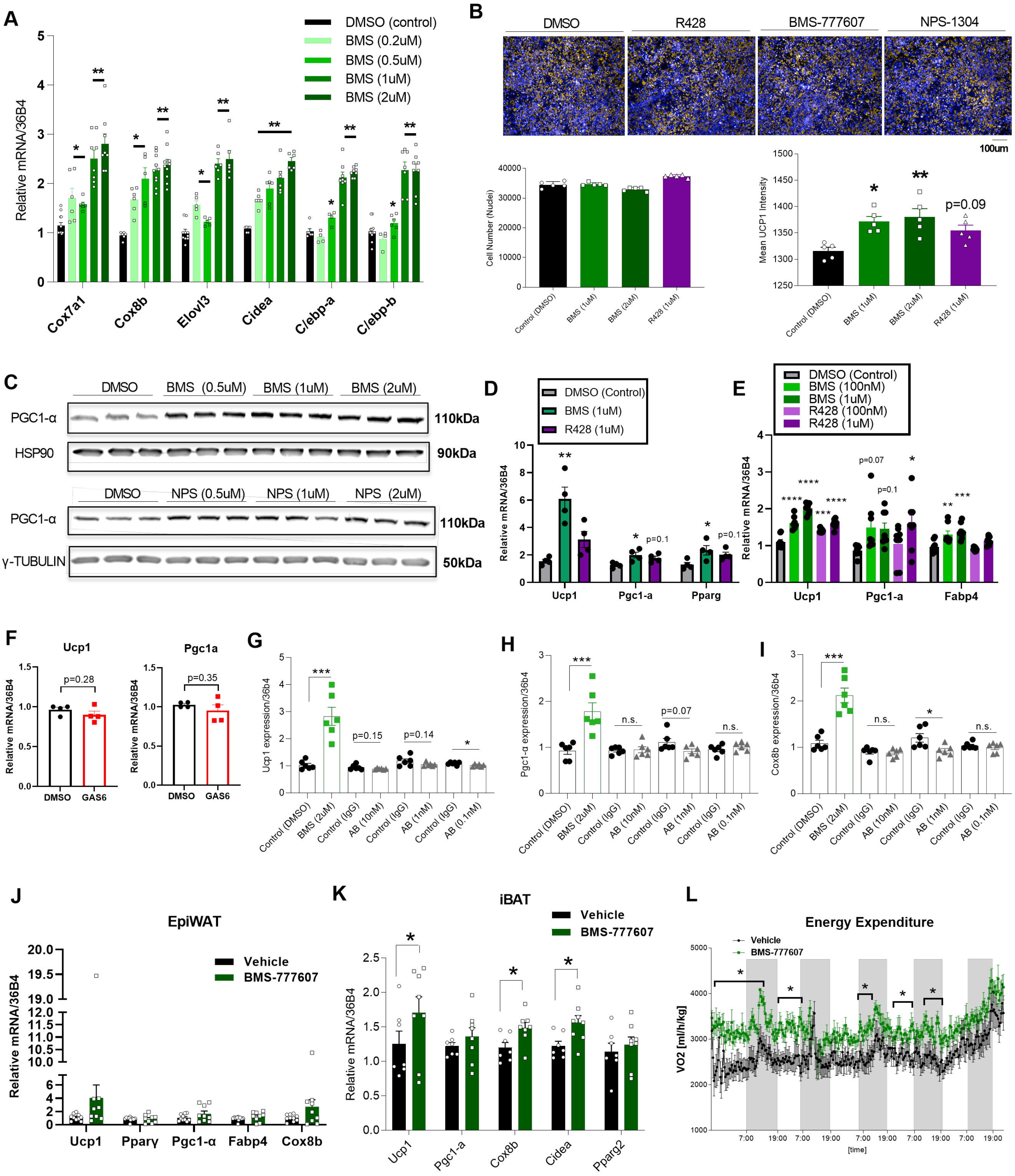

Figure S2: A) Gene expression (qRT-PCR) analysis. Representative graph of 4-6 independent experiments showing mRNA expression of brown-enriched genes in isoproterenol-stimulated mature iBAs after treatment with increasing concentrations of the AXL inhibitor BMS-777607 (BMS – green color) (n=6/group). * denotes significance compared to control. B) High-throughput microscopic fluorescent analysis. Representative

fluorescent images showing iBAs differentiation based on lipid droplet staining (LD540/yellow: lipid droplets, blue/A497: Hoechst/Nuclei) (up), (low) representative graphs of 3 independent experiments showing number of nuclei (left) and UCP1 intensity/cell (right) (n=6/group). * denotes significance compared to control. C) Representative western blots of 2 independent experiments demonstrating protein expression of PGC1-α in iBAs after treatment with increasing dosages of the AXL inhibitors BMS (upper) and NPS-1034 (lower). D) Gene expression (qRT-PCR) analysis. mRNA expression of Ucp1, Pgc1-α and Pparγ in isoproterenol-stimulated mature white immortalized adipocytes (iWAs) after treatment with the AXL inhibitors BMS-777607 (BMS – green color) or R428 (purple) (n=4/group). * denotes significance compared to control. E) Gene expression (qRT-PCR) analysis. mRNA expression of UCP1, PGC1-α and FABP4 in brown-like hMADS after treatment with the AXL inhibitors BMS-777607 (BMS – green color) or R428 (purple) (n=8/group). * denotes significance compared to control. F) Gene expression (qRT-PCR) analysis. mRNA expression of Ucp1, Pgc1-α in mature iBAs in response to GAS6 treatment (n=4/group). G-I) Gene expression (qRT-PCR) analysis. mRNA expression of Ucp1 (G), Pgc1-α (H), and Cox8b (I) in mature iBAs in response to agonistic anti-AXL receptor antibody treatment (n=6/group). J-L) Two-week daily (food-mixed) administration of the AXL inhibitor BMS-777607 or vehicle control in C57Bl6 DIO mice. J-K) Gene expression (qRT-PCR) analysis. mRNA expression of thermogenesis-associated genes in epiWAT (J) and iBAT (K) (Vehicle n=10; BMS-777607 n=9). L) VO_2 metabolic cage measurements two weeks after daily administration of BMS-777607 or vehicle control (n=6/group). For all graphs, results are presented as average ± SEM. * $p<0.05$, ** $p<0.01$, *** $p<0.001$. For two group comparisons (F, J, K) unpaired two-tailed t-test was performed, for three or more group comparisons (A, B, D, E, G, H, I) one-way ANOVA was performed (Tukey test was applied to correct for multiple comparisons).

Supplementary Figure 3

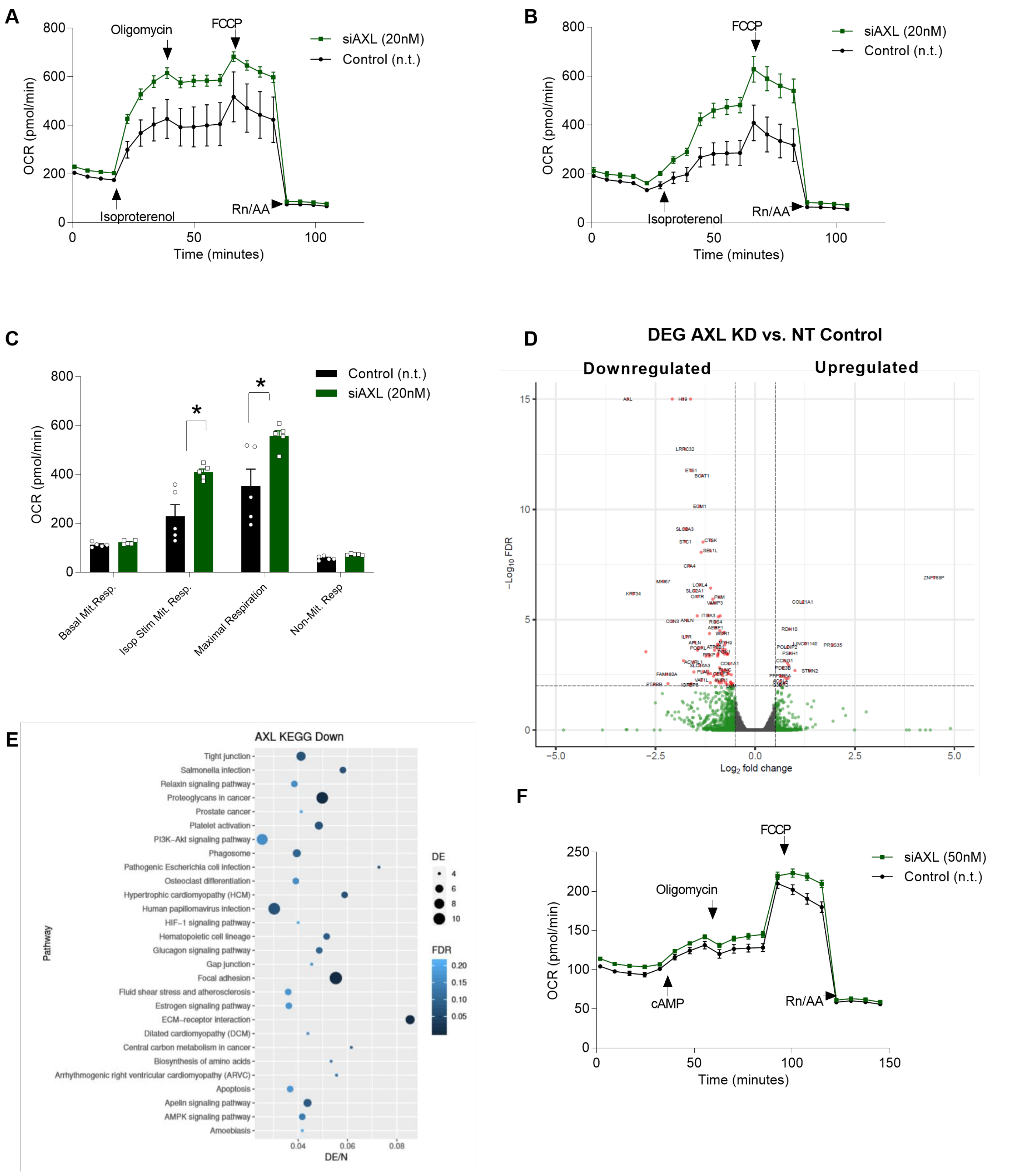

Figure S3: siRNA-mediated knockdown of AXL receptor enhances brown adipocyte functionality. A) Measurements of oxygen consumption rate (OCR) for the calculations of basal, isoproterenol-stimulated,

maximal, uncoupled and non-mitochondrial respiration of mature iBAs upon siRNA-mediated knockdown of AXL receptor (n=5/group). B) Measurements of oxygen consumption rate (OCR) for the calculations of basal, isoproterenol-stimulated, maximal and non-mitochondrial respiration of mature iBAs upon siRNA-mediated knockdown of AXL receptor (n=5/group). C) Calculations of basal, cAMP-stimulated, maximal, uncoupled, coupled and non-mitochondrial respiration in iBAs upon siRNA-mediated knockdown of AXL receptor, based on OCR measurements (n=5/group). (D-F) siRNA-mediated knockdown in brown hMADS. D) Volcano plot demonstrating the differentially expressed genes between si-AXL and non-targeting (NT) control in brown hMADS. E) KEGG pathway enrichment analysis for downregulated genes upon siRNA-mediated knockdown of AXL in brown hMADS. F) Measurements of oxygen consumption rate (OCR) for the calculations of basal, cAMP-stimulated, maximal, uncoupled, coupled and non-mitochondrial respiration in brown hMADS upon siRNA-mediated knockdown of AXL receptor (n=5/group). For all graphs, results are presented as average ± SEM. * $p<0.05$, ** $p<0.01$, *** $p<0.001$. For two group comparisons (C) unpaired two-tailed t-test was performed, for time-course datasets two-way ANOVA was performed.

Supplementary Figure 4

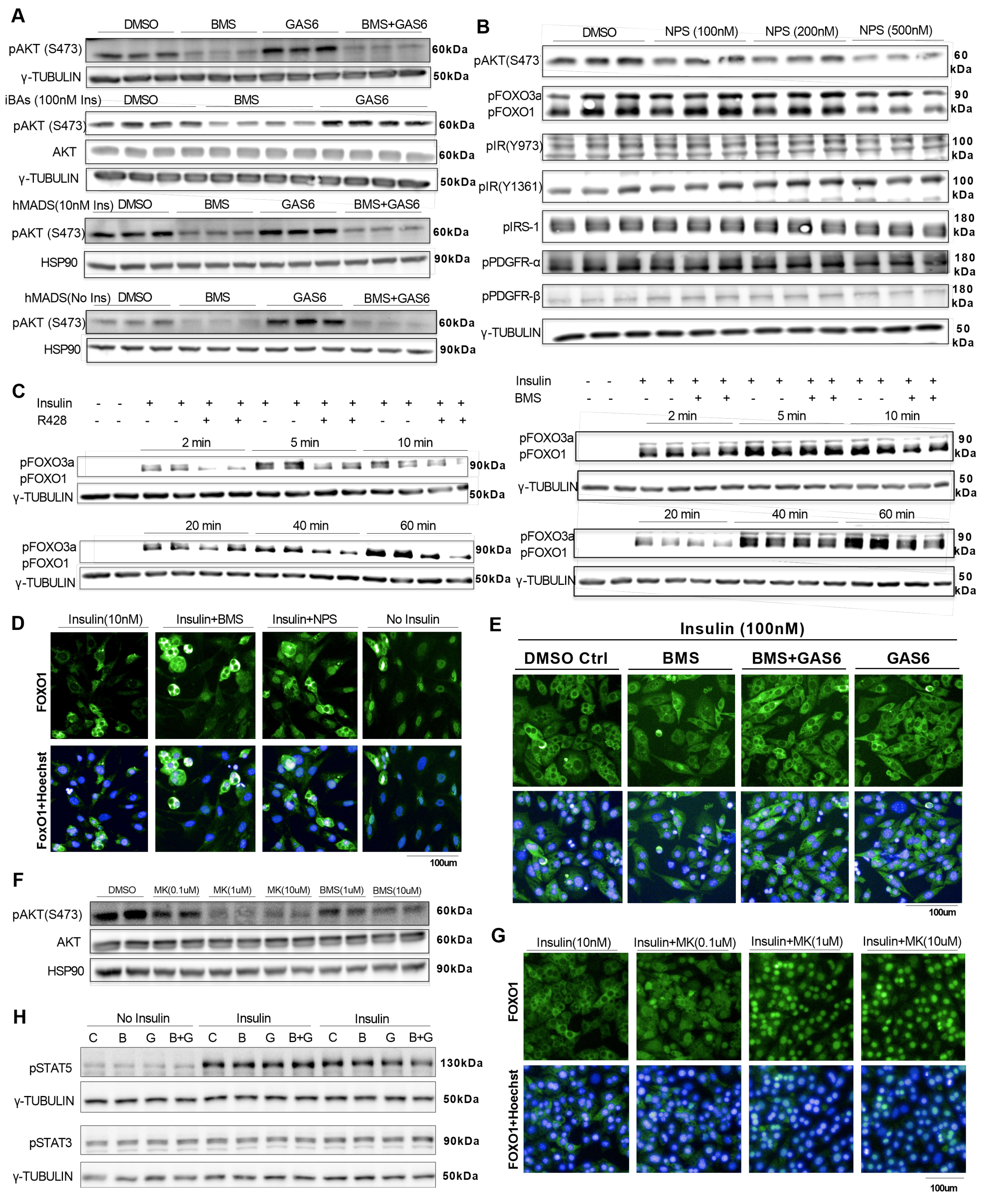

Figure S4: A) Representative western blots of three independent experiments demonstrating protein levels of phosphorylated AKT upon acute treatment with the AXL inhibitor BMS-777607 (BMS) and the AXL receptor

agonist GAS6 in unstimulated and insulin-stimulated mature iBAs (upper) and hMADS (lower). B) Representative western blots of 2 independent experiments depicting protein levels of phosphorylated AKT, FOXO, IR, IRS-1, PDGFR-α and PDGFR-β after acute treatment with NPS-1034 in insulin-stimulated mature iBAs. C) Representative western blot analysis of 2 independent experiments depicting protein levels of phosphorylated FOXO1 upon acute treatment with the AXL inhibitors R428 (1uM) (left) and BMS-777607 (10uM) (right) in insulin-stimulated mature iBAs for several time points after insulin stimulation. D) Representative immunofluorescent images (A488/green: FOXO1, blue/A497: Hoechst/Nuclei) of 3 independent experiments showing the cytoplasmic and nuclear localization of FOXO1 in non-stimulated and insulin-stimulated mature iWAs upon treatment with AXL receptor pharmacological inhibitors BMS-777607(BMS) or NPS. Insulin-stimulated cells were treated with 10nM of insulin. E) Representative immunofluorescent images (A488/green: FOXO1, blue/A497: Hoechst/Nuclei) of three independent experiments showing the cytoplasmic and nuclear localization of FOXO1 in high-dosage insulin-stimulated mature iBAs upon treatment with AXL receptor pharmacological inhibitor BMS-777607(BMS) or ligand GAS6 or combination of BMS+GAS6. Insulin-stimulated cells were treated with 100nM of insulin. F) Representative western blot analysis of 2 independent experiments depicting protein levels of phosphorylated AKT (pAKT) upon acute treatment with increasing concentrations of the AKT inhibitor MK-2206. G) Representative immunofluorescent images (A488/green: FOXO1, blue/A497: Hoechst/Nuclei) of 3 independent experiments showing the cytoplasmic and nuclear localization of FOXO1 in insulin-stimulated mature iBAs upon treatment with increasing dosages of the AKT inhibitor MK-2206. Cells were stimulated with 10nM of insulin. H) Representative western blot analysis of 2 independent experiments depicting protein levels of phosphorylated STAT3 (pSTAT3) and STAT5 (pSTAT5) in non-stimulated and insulin-stimulated iBAs in response to acute treatment with AXL receptor pharmacologic inhibitor BMS-777607 (B), ligand GAS6 (G), or a combination of both (B+G).

Supplementary Figure 5

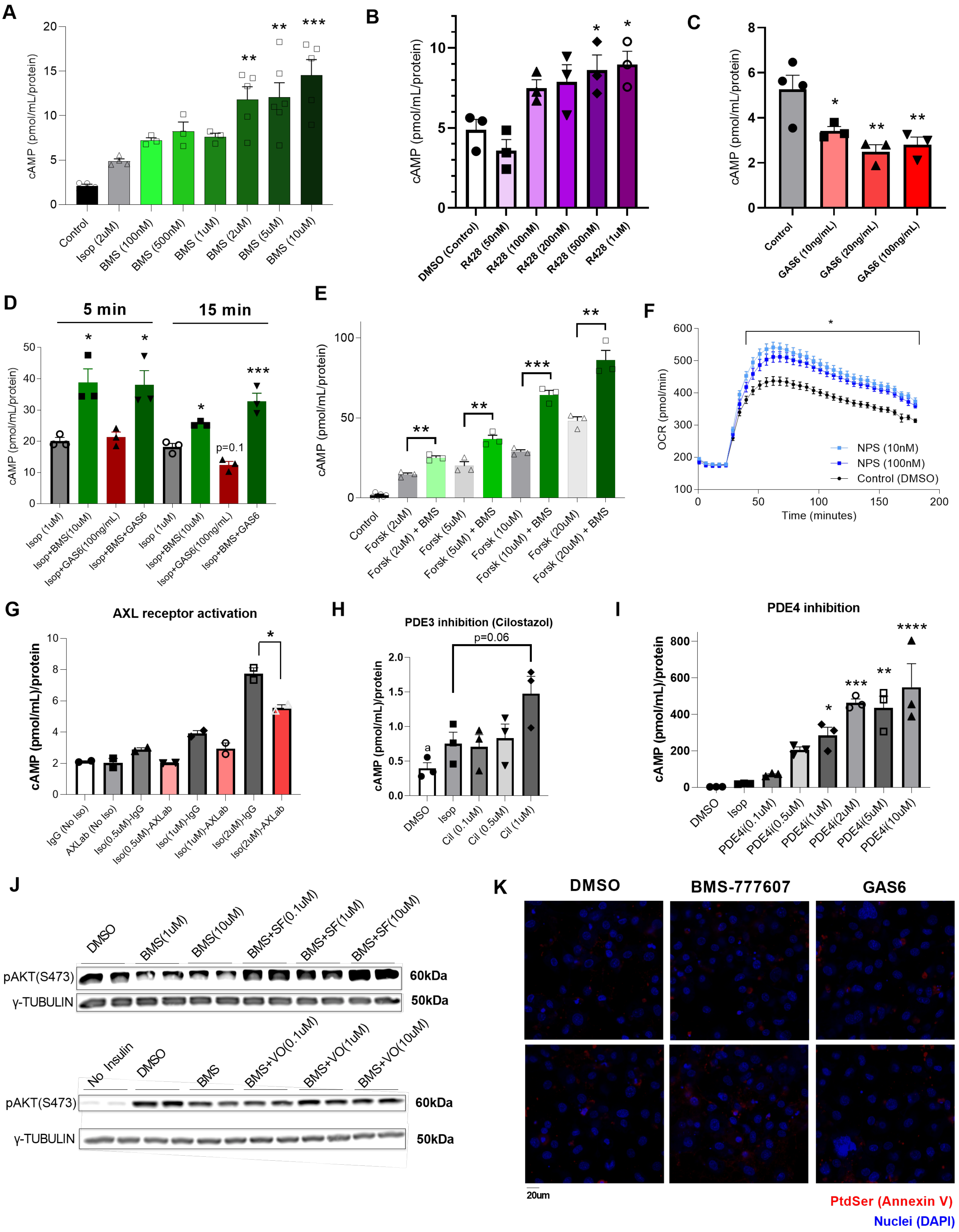

Figure S5: A-C) Intracellular cAMP levels after acute isoproterenol stimulation upon simultaneous treatment with increasing dosages of BMS-777607 (Ctrl n=4; Isop n=4; BMS 2uM n=5; BMS 5uM n=6; BMS10uM n=5; all others, n=3/group) (A), R428 (n=3/group) (B), or GAS6 (n=3/group) (C). cAMP was measured at 15 min after isoproterenol/compound stimulation. * denotes significance compared to isoproterenol control. D) Intracellular cAMP levels after acute isoproterenol stimulation upon simultaneous treatment with BMS, GAS6 or BMS+GAS6. cAMP was measured at time points 5 and 15 min after isoproterenol/compound stimulation (n=3/group). * denotes significance compared to control within the same time-point. E) Intracellular cAMP levels after acute (15 min) stimulation with increasing dosages of forskolin in the presence or absence of the AXL inhibitor BMS (n=3/group). * denotes significance compared to forskolin control. F) OCR Seahorse measurements in compound-pre-treated acutely isoproterenol-stimulated iBAs upon concomitant treatment with the AXL inhibitor NPS (n=5/group). Arrow indicates the time of injection of isoproterenol and compound. G) Intracellular cAMP levels after increasing doses of acute isoproterenol stimulation upon simultaneous treatment with the anti-AXL monoclonal agonistic antibody (n=3/group) H-I) Intracellular cAMP levels at the 15min timepoint after acute isoproterenol stimulation upon simultaneous treatment with incremental doses of the PDE3 inhibitor Cilostazol (H) or a PDE4 inhibitor (I) (n=3/group). * denotes significance compared to control. J) Representative western blot analysis of 2 independent experiments depicting protein levels of phosphorylated AKT (pAKT) upon acute treatment with pharmacological inhibitor of AXL receptor (BMS) and PTEN inhibitors SF1670 (SF) or bpV(HOpic) (VO). K) Annexin V staining to detect Ptd-Ser in mature iBAs in response to treatment with AXL receptor pharmacological inhibitor BMS or its ligand GAS6. PtdSer has been stained in red and nuclei have been stained in blue (DAPI). For all graphs, results are presented as average ± SEM. ** $p<0.05$, ** $p<0.01$, *** $p<0.001$. For two group comparisons (G) unpaired two-tailed t-test was performed, for three or more group comparisons (A, B, C, D, E, H, I) one-way ANOVA was performed (Tukey test was applied to correct for multiple comparisons), and for time-course datasets (F) two-way ANOVA was performed.

Supplementary Figure 6

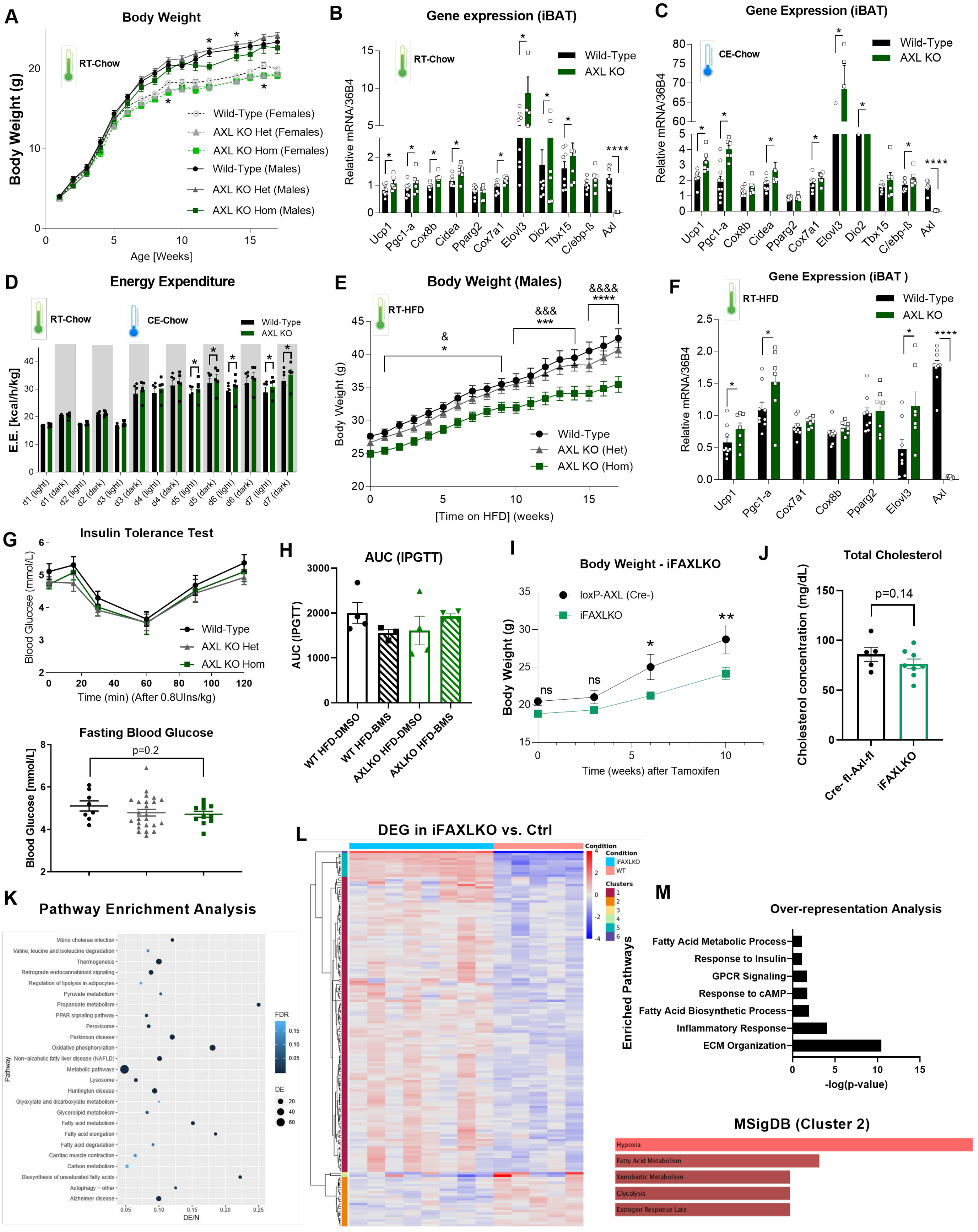

Figure S6: A) Body weight curves of *Axl* KO homozygous (AX KO Hom), *Axl* KO heterozygous (AXLKO Het) and Wild-Type male and female littermates under chow diet during the first weeks of age. (n=12, 25, 14 for the males; and n=10, 20, 13 for the females, respectively). B-C) Gene expression (qRT-PCR) analysis. mRNA expression of brown fat thermogenesis-enriched genes in iBAT of *Axl* KO and WT littermate control male chow diet-fed mice under room temperature (B) or two-day cold exposure (C.E.) (C) conditions (WT n=8; *Axl* KO n=6). D) Energy expenditure (calculated based on VO_2) metabolic cage measurements in *Axl* KO and WT littermate control chow diet-fed mice under room temperature and cold-exposure (n=6/group). Temperature conditions are indicated above the graph. E) Body weight curves of *Axl* KO homozygous (AXLKO Hom), *Axl* KO heterozygous (AXLKO Het) and Wild-Type male littermates under HFD challenge. HFD was initiated at 8 weeks of age, mice were acclimatized in room-temperature conditions (n=22, 27, 20; respectively). F) Gene expression (qRT-PCR) analysis. mRNA expression of brown fat thermogenesis-enriched genes in iBAT of *Axl* KO and WT littermate control male HFD-fed mice under room temperature conditions (n=8 for WT, n=6 for AXLKO). G) Insulin tolerance test (WT n=8; AXLKO Het n=24; *Axl* KO Hom n=11) (upper) and fasting blood glucose (WT n=8; AXLKO Het n=24; *Axl* KO Hom n=11) (lower) in *Axl* KO homozygous (AXLKO Hom), *Axl* KO heterozygous (AXLKO Het) and Wild-Type male littermates under HFD challenge. H) Area Under the Curve (AUC) calculated from IPGTT in *Axl* KO and WT littermate male mice in response to treatment with the pharmacological AXL receptor inhibitor BMS-777607 or DMSO control (WT HFD-BMS n=3; all other groups n=4). I) Body weight curves of iFAXLKO and littermate control (AXL-floxed Cre-) female mice in response to HFD challenge. HFD was initiated at 8 weeks of age, mice were acclimatized in standard room-temperature conditions (n=11/group). J) Total cholesterol concentration measurements in plasma of *Axl* KO and WT littermate control HFD-fed mice (WT n=5; *Axl* KO n=8). K) Pathway Enrichment Analysis from RNA Sequencing analysis of differentially expressed genes in iBAT depots of *Axl* KO vs. WT littermate control male mice under HFD and room-temperature ambient conditions. L) Heatmap demonstrating differentially expressed genes in iBAT depots of iFAXLKO as compared to WT littermate female mice under HFD and room-temperature ambient conditions (n=5/group). M) Overrepresentation pathway analysis and Enrichr pathway analysis based on differentially upregulated genes in iBAT depots of iFAXLKO vs. WT littermate control female mice (for RNA-Seq, n=8 for iFAXLKO, n=5 for Axl-floxed Cre-). For all graphs, results are presented as Average ± SEM. * p-value<0.05, ** p-value<0.01, *** p-valued<0.001, **** p-value<0.0001 (for WT vs. AXL KO Hom). $^{\&}$ p-value<0.05, $^{\&\&}$ p-value<0.01, $^{\&\&\&}$ p-value<0.001, $^{\&\&\&\&}$ p-

value<0.0001 (for AXLKO Het vs. AXLKO Hom). * p<0.05, **p<0.01, ***p<0.001. Two-group comparisons (B, C, F, J): unpaired two-tailed t-test; three or more group comparisons (G, H): one-way ANOVA (Tukey test was applied to correct for multiple comparisons); time-course datasets (A, D, E, G, I) two-way ANOVA.

Supplementary Table 1

Receptor / Compound	Axl	RON	Met	Tyro3	Mer
**BMS-777607**	1.1nM	1.8nM	3.9nM	4.3nM	14nM
**R428**	14nM	-	-	50- to 100-fold less selective	50- to 100-fold less selective
**NPS-1034**	10.3nM	-	48nM	-	-

Supplementary Table 1: IC50 values of three pharmacological AXL receptor inhibitors for AXL receptor and other receptor tyrosine kinases. Dash (-) indicates a complete lack of cross-reactivity.

Supplementary References

1 Fagerberg, L. *et al.* Analysis of the Human Tissue-specific Expression by Genome-wide Integration of Transcriptomics and Antibody-based Proteomics. *Mol Cell Proteomics* **13**, 397-406, doi:10.1074/mcp.M113.035600 (2014).
