# Supplementary material for: Inhibition of AXL receptor tyrosine kinase enhances brown adipose tissue functionality in mice
Source: Nat Commun. 2023 Jul 13;14:4162. doi: 10.1038/s41467-023-39715-8 (PMC10344962; doi:10.1038/s41467-023-39715-8)
Supplement: Supplementary file 3 — Reporting Summary [file 41467_2023_39715_MOESM3_ESM.pdf]

nature portfolio

Corresponding author(s): Christian Wolfrum

Last updated by author(s): Jun 25, 2023

Reporting Summary

Nature Portfolio wishes to improve the reproducibility of the work that we publish. This form provides structure for consistency and transparency in reporting. For further information on Nature Portfolio policies, see our Editorial Policies and the Editorial Policy Checklist.

Statistics

For all statistical analyses, confirm that the following items are present in the figure legend, table legend, main text, or Methods section.

n/a	Confirmed	
☐	☒	The exact sample size (n) for each experimental group/condition, given as a discrete number and unit of measurement
☐	☒	A statement on whether measurements were taken from distinct samples or whether the same sample was measured repeatedly
☐	☒	The statistical test(s) used AND whether they are one- or two-sided<br>*Only common tests should be described solely by name; describe more complex techniques in the Methods section.*
☒	☐	A description of all covariates tested
☐	☒	A description of any assumptions or corrections, such as tests of normality and adjustment for multiple comparisons
☐	☒	A full description of the statistical parameters including central tendency (e.g. means) or other basic estimates (e.g. regression coefficient) AND variation (e.g. standard deviation) or associated estimates of uncertainty (e.g. confidence intervals)
☐	☒	For null hypothesis testing, the test statistic (e.g. F, t, r) with confidence intervals, effect sizes, degrees of freedom and P value noted<br>*Give P values as exact values whenever suitable.*
☒	☐	For Bayesian analysis, information on the choice of priors and Markov chain Monte Carlo settings
☒	☐	For hierarchical and complex designs, identification of the appropriate level for tests and full reporting of outcomes
☒	☐	Estimates of effect sizes (e.g. Cohen's d, Pearson's r), indicating how they were calculated

Our web collection on statistics for biologists contains articles on many of the points above.

Software and code

Policy information about availability of computer code

Data collection	Western blots were visualized by the Image Quant system (GE Healthcare Life Sciences) or by Sally Sue (ProteinSimple, Bio-Techne). Quantitative PCR was performed using ViiA7 realtime PCR system (Applied Biosystems). Image acquisition for immunoflorescence was done by Operetta automated microscope (Perkin Elmer). Biochemical colorimetric assays or luminiscence was detected by SynergyMx plate reader (BioTek). Cellular respiration was monitored by XF96 Extracellular Flux Analyzer (Agilent Seahorse). Indirect calorimetry measurement was performed by automated home cage phenotyping Phenomaster (TSE-systems). Live mice body composition was measured with a magnetic resonance imaging technique (EchoMRI130, Echo Medical Systems). Tissue section were visualized by Axiophot microscope equipped with AxioCam MR (Zeiss).
Data analysis	Quantification of Western blots was done by ImageJ v 1.53e (NIH). Real-time PCR was analyzed by ViiA7 Ruo v1.2.3 (ThermoFisher). Immunofluorescence image analysis was performed either by Harmony v3.5 (Perkin Elmer). Colorimetric assays were analyzed by Gen5 v3.08 (BioTek). Cell respiratory analysis was done by Wave 2.6.0 (Agilent Seahorse). Energy expenditure analysis was performed by Phenomaster software v5.6.5 (TSE-systems). Fat and lean mass was analyzed using Echo MRI 14 software. Statistical analysis was performed by GraphPad Prism 9.

For manuscripts utilizing custom algorithms or software that are central to the research but not yet described in published literature, software must be made available to editors and reviewers. We strongly encourage code deposition in a community repository (e.g. GitHub). See the Nature Portfolio guidelines for submitting code & software for further information.

Data

Policy information about availability of data

All manuscripts must include a data availability statement. This statement should provide the following information, where applicable:

- Accession codes, unique identifiers, or web links for publicly available datasets
- A description of any restrictions on data availability
- For clinical datasets or third party data, please ensure that the statement adheres to our policy

RNA-Sequencing datasets (hMADS transcriptomic datasets after ablation of AXL receptor, iBAT transcriptomic datasets of Axl KO vs. WT mice and iBAT transcriptomic datasets of iFAXLKO vs. WT mice) used in this study have been deposited and are available in GEO under accession number GSE231471.

Human research participants

Policy information about studies involving human research participants and Sex and Gender in Research.

Reporting on sex and gender	N/A
Population characteristics	N/A
Recruitment	N/A
Ethics oversight	N/A

Note that full information on the approval of the study protocol must also be provided in the manuscript.

Field-specific reporting

Please select the one below that is the best fit for your research. If you are not sure, read the appropriate sections before making your selection.

[x] Life sciences [ ] Behavioural & social sciences [ ] Ecological, evolutionary & environmental sciences

For a reference copy of the document with all sections, see nature.com/documents/nr-reporting-summary-flat.pdf

Life sciences study design

All studies must disclose on these points even when the disclosure is negative.

Sample size	Sample size was determined based on availability of mice and their specific genotype (homozygous, heterozygous, or wild-type). For all other in vitro and in vivo experiments, sample size was determined based on previous experiments in our lab and similar studies reported in the literature. (Balaz et al. 2019 Cell Metab; Sun et al. 2018 Nat Med; Sun et al. 2020 Nature).
Data exclusions	For in vitro and in vivo experiments, no samples and individuals were excluded from analyses.
Replication	Unless stated otherwise, all cell culture experiments were independently reproduced 2-4 times as indicated. All animal experiments were repeated independently 2 or 3 times as indicated. In particular, whole-body knockout findings were reproduced in three independent cohorts and tissue-specific knockout findings were reproduced in two independent cohorts. Pharmacological studies in wild-type mice were reproduced in two independent cohorts. Pharmacological + knockout studies were performed in a single cohort however mice were obtained from different litters to exclude this co-variate.
Randomization	For each study, all animals (littermates) were randomly allocated into experimental groups. Regarding cell culture experiments, culture wells were randomly assigned to treatments in each independent round of experiment to avoid any plate effect.
Blinding	If not stated otherwise, investigators were blinded to allocation of mice to sample groups during experiments and data analysis. Metabolic phenotyping of mice was performed by technicians, unaware of the study design.

Reporting for specific materials, systems and methods

We require information from authors about some types of materials, experimental systems and methods used in many studies. Here, indicate whether each material, system or method listed is relevant to your study. If you are not sure if a list item applies to your research, read the appropriate section before selecting a response.

Materials & experimental systems

n/a	Involved in the study
☐	☒ Antibodies
☐	☒ Eukaryotic cell lines
☒	☐ Palaeontology and archaeology
☐	☒ Animals and other organisms
☒	☐ Clinical data
☒	☐ Dual use research of concern

Methods

n/a	Involved in the study
☒	☐ ChIP-seq
☒	☐ Flow cytometry
☒	☐ MRI-based neuroimaging

Antibodies

Antibodies used

UCP1 (ThermoFisher, Cat# PA1-24894, RRID: AB_2241459), γ-TUBULIN (Sigma-Aldrich, Cat# T-5326, RRID: AB_532292), HSP90 (Cell Signaling, Cat# 4877, RRID: AB_2233307), phospho-CREB Ser133 (Cell Signaling, Cat# 9198, RRID: AB_2561044), phospho-PKA substrates (Cell Signaling, Cat# 9624, RRID:AB_331817), phospho-AKT Thr308 (Cell Signaling, Cat# 13038, RRID: AB_2629447), phospho-AKT Ser473 (Cell Signaling, Cat# 4060, RRID: AB_2315049), phospho-Foxo1 (Thr24)/FoxO3a (Thr32) (Cell Signaling, Cat# 9464, RRID:AB_329842), FOXO1 (Cell Signaling, Cat# 2880, RRID:AB_2106495), anti-mouse HRP secondary (Merck, Cat# 401253, RRID: AB_437779), anti-rabbit HRP secondary (Merck, Cat#401393, RRID: AB_10683386), anti-mouse Alexa Fluor 488 secondary (Thermo Fisher, Cat# A-11029, RRID:AB_2534088), anti-rabbit Alexa Fluor 488 secondary (Thermo Fisher, Cat# A-21206, RRID:AB_2535792), AXL (Abcam, Cat# ab215205, RRID: AB_2924328), AXL (ThermoFisher, Cat# PA5-106118, RRID: AB_2853927), PGC1-a (Millipore Sigma, Cat# AB3242, RRID: AB_2268462), phospho-Insulin Receptor (Y972) (Abcam, Cat#ab5678, RRID: AB_305045), phospho-Insulin Receptor (Y1361) (Abcam, Cat#ab60946, RRID: AB_943587), phospho-IRS1 (ThermoFisher, Cat#PA5-114593, RRID: AB_2899229), phospho-AXL (,Tyr702) (ThermoFisher, Cat# PA5-64862, RRID: AB_2662770), phospho-STAT3 (Ser727) (Cell Signaling, Cat#9134, RRID: AB_331589), phospho-STAT5 (Tyr694) (Cell Signaling, Cat#4322, RRID: AB_10544692), phospho-PDGFRA (Y742) (Abcam, Cat#ab5452, RRID: AB_304899), phospho-PDGFRB (Y857) (Abcam, Cat#ab62367, RRID: AB_2162635)

Validation

Primary antibodies used in this study were validated either by siRNA mediated knockdown or whole-body/tissue specific genetic knockout mouse models of target protein (AXL), or with the use of positive controls (insulin treatment for validation of phospho-AKT Thr308 or Ser473 or FOXO1 or phospho-FOXO1, GAS6 and pharmacological inhibitor treatment for validation of phospho-AXL, isoproterenol treatment for phospho-CREB Ser133 and phospho-PKA substrates antibodies).
Other antibodies such as phospho-IR (Y973, Y1361), phospho-IRS1 (insulin), phospho-PDGFRA and phospho-PDGFRB, phospho-AKT Thr308 and phospho-Foxo1 Thr24 (insulin) were previously validated in laboratory in other experiments including positive control (validation method indicated in brackets). In addition, several widely used and accepted antibodies, which were validated by manufacturers and other research groups were used (UCP1, PGC1-a, γ-TUBULIN, HSP90, phospho-STAT3, phospho-STAT5, phospho-PDGFRA, phospho-PDGFRB).

Eukaryotic cell lines

Policy information about cell lines and Sex and Gender in Research

Cell line source(s)

hMADS cells - Dr. Ez-Zoubir Amri, University of Nice, France (Elabd et al., 2009)
murine immortalized brown adipocytes (iBAs) - Prof. Ronald C. Kahn, Harvard University, Boston, USA (Klein et al., 2002)
murine immortalized white adipocytes (iWAs) - (Kovsan et al., 2009)
A549 human lung carcinoma cell line was purchased from ATCC (Cat.No. CCL-185)

Authentication

hMADS, iWA, and iBA cell lines are commonly used to study white and brown adipocyte physiology and function, and were extensively tested in our previous studies as well as by other groups. These cell lines were authenicated by PCR assay using species-specific primers to determine expression level of key thermogenic protein UCP1. A549 cells were purchased from ATCC.

Mycoplasma contamination

All cell lines used in the study were repeatedly tested negative for mycoplasma contamination every three months.

Commonly misidentified lines
(See ICLAC register)

No commonly misidentified cell lines were used

Animals and other research organisms

Policy information about studies involving animals; ARRIVE guidelines recommended for reporting animal research, and Sex and Gender in Research

Laboratory animals

Mus musculus, strains C57BL6/N (Charles River), Axl null mice, Axl floxed, Axl floxed x Adiponectin CreERT2 mice, all adult males and females (8-35 weeks). For more details, please see methods section or figure legends.

Wild animals

Study did not involve wild animals.

Reporting on sex

For pharmacological studies, we used male mice. For whole-body knockout studies we used both males and female mice. For tissue-specific knockout studies we used female mice, due to the availability of the appropriate genotype.

Field-collected samples	Study did not involve samples collected from the field
Ethics oversight	All animal procedures were approved by the Veterinary office of the Canton of Zürich. Health status of all mouse lines was regularly monitored according to FELASA guidelines.

Note that full information on the approval of the study protocol must also be provided in the manuscript.
